# “I feel like people would look at me like I’m dirty or like I’m a thief:” a qualitative exploration of interpersonal and intrapersonal experiences that influence recovery among adults at risk of opioid-related overdose

**DOI:** 10.3389/fpubh.2025.1464075

**Published:** 2025-02-28

**Authors:** Hannah S. Szlyk, Sara Jones, Jordan Michener, Zhuoran Zhang, Nina Kaiser, Heidi Holtz, Melody Rachel Konadu Frempong, Husain Lateef, William Hutson, Patricia Cavazos-Rehg

**Affiliations:** ^1^Department of Psychiatry, Washington University School of Medicine, St. Louis, MO, United States; ^2^Barnes Jewish College, Goldfarb School of Nursing, St. Louis, MO, United States; ^3^Brown School at Washington University in St. Louis, St. Louis, MO, United States

**Keywords:** opioid use, stimulant use, interpersonal, intrapersonal, qualitative methods

## Abstract

**Objective:**

Due to the nature of early termination of treatment and the transient lifestyles of many people who use substances, many providers are unaware of the factors that may have contributed to their client’s engagement in treatment. Treatment providers and researchers need a better understanding of intrapersonal and interpersonal factors that impact recovery from the clients’ perspective. This study uses qualitative methods to explore these factors.

**Methods:**

Eligible participants were adults who had used opioids and/or stimulants and were receiving treatment at a consenting facility. Participants completed one-month follow-up interviews after using a mobile health intervention. Transcripts were analyzed using deductive thematic analyses. Two research staff members coded the transcripts independently. The third coder identified coding discrepancies.

**Results:**

Out of 24 participants, most were middle-aged, and the majority identified as female and as white. Half of participants (*n* = 12) shared that they have recovery-related worries, predominantly about finances and finding work after treatment. Twenty participants (*n* = 20) discussed how they currently take care of their daily needs. All participants shared proactive factors that support recovery, specifically seeking motivation from loved ones. Conversely, many participants shared how they had strained familial relationships when using substances. Twenty participants reported experiencing the theme of stigma due to their substance use, specifically by family members and providers.

**Conclusion:**

Most participants are concerned about their financial future; the respective treatment facilities help to meet current basic needs. Most participants have relationships with family members and identify them as a source of support in recovery. Overall, many individuals in recovery have the tools to address interpersonal and intrapersonal challenges. Patients may still benefit from assistance planning for life post-treatment.

## 1 Introduction

Overdose death related to opioid use is major public health issue in the US, with numbers increasing following the COVID-19 pandemic (1). Similarly, drug overdose deaths in Missouri have become a widespread health problem. Drug overdose is the number one leading cause of death in Missouri among adults aged 18–44 (2). In 2022, it was estimated that 778,000 Missouri adolescents and adults experienced substance use disorders (SUDs) during the past year (3).

Funding to address opioid-related overdose (4) has been beneficial in extending access to treatment. Yet, in 2022 only 55% of people in need of opioid use disorder treatment received any within the past year (5). Of those receiving treatment, many leave prematurely. For example, a 2021 study found that 64% of people receiving medication for opioid use disorder (MOUD) in outpatient settings leave treatment prematurely (6). A systematic review estimates that the MOUD retention rate is 57% at 12 months, and 38% at 3 years (7). In Missouri, these 12 months retention rates are much lower than the national average, at less than 10% in 2022 (8). Less is known about retention rates for non-MOUD treatment facilities.

Various psychosocial factors also complicate treatment and recovery outcomes. Being unemployed and uninsured makes it difficult for people with SUDs to continue maintenance of medical care (9) and many state-level Medicaid programs do not provide comprehensive coverage of issues related to SUDs (10). A lack of perceived social support has also been found to be related with poorer mental health and stigma among people with SUDs (11)—both factors that can negatively impact recovery outcomes (12, 13). Additionally, healthcare providers face their own challenges when treating people with SUDs. A 2024 systematic review found that physicians most often reported a lack of institutional support (81.2%), a lack of personal cognitive capacity to manage complexities of care (73.5%), a lack of specific skills (73.9%), and inadequate knowledge (72%) as barriers to adequately treating substance use (14).

Due to the nature of early termination of treatment and the transient lifestyles of many people who use substances, many providers may not know why their clients made this decision or which factors may have impeded treatment engagement at all. For example, Trujols and colleagues (15) found a significant difference between patient-reported and clinician-reported improvement of methadone maintenance treatment, as patients documented greater improvement in their condition versus the assessment by the clinical care providers. Similarly, Mitchell-Foster et al. (16) reported a disconnect in how pregnant women who use substances and providers recognized stigmatizing situations in the delivery of care. Thus, this likely disconnect may only contribute to the disparity in treatment engagement (17). Ideally, treatment providers and addictions researchers need a more in-depth understanding of intrapersonal (e.g., one’s own thoughts and self-reflection) and interpersonal (e.g., communication and interaction with others) factors that impact recovery that are in the words of the client (18–20).

A recovery-oriented approach emphasizes the importance of integrating the experiences and voices of people who use substances in the planning and the delivery of care (21, 22). Driven by this approach, recovery support services are non-clinical and medication-based treatments and may include peer support, mutual aid groups, and community centers that offer support with attending to risk factors that can threaten recovery (e.g., housing instability, unemployment, lack social support, food insecurity) (23–25). Emerging evidence demonstrates that recovery-oriented services that address these factors, especially stable housing and social support, may promote better recovery outcomes (26, 27). In the recent years, recovery community centers (RCCs) have expanded rapidly across the U.S. and have emerged as a social recovery hub that increases recovery capital such as employment and housing (28). As more addiction services adopt a recovery-oriented approach, there is a growing need to formally incorporate peer support into recovery support services (29). Peer recovery support services, delivered by individuals with lived experience, aim to strengthen social connectedness, enhance quality of life, improve education and housing, and reduce involvement with the criminal justice system (30).

The study aimed to explore intrapersonal and interpersonal factors that support and impede recovery from substance use disorders based on the perspectives of adults living in Missouri. Findings may offer providers working in Missouri and similar states a nuanced understanding of clients’ barriers and strengths contributing to recovery, which may lead to more collaborative treatment planning, the integration of recovery services, and, overall, better recovery outcomes.

## 2 Methods

### 2.1 Participants and study design

In this study, we utilized follow-up qualitative data from individuals who participated in a supplementary digital intervention known as uMAT-R. This intervention was designed to provide support for adults who are in the process of recovering from SUD(s). uMAT-R (pronounced “you matter”) is a digital tool designed to support unique challenges, preferences, and needs related to recovery, to ultimately improve recovery outcomes for people with SUDs. The uMAT-R digital intervention has several key components, including (1) an in-app human coach (e-coach) trained in motivational interviewing who provides near real-time feedback and support to help clients stay motivated towards recovery and navigate uMAT-R, (2) psychoeducation modules based on cognitive-behavioral approaches for the treatment of SUDs and co-occurring mental health conditions, and (3) a community directory to help participants secure basic needs (e.g., housing, food pantries) and access recovery supports (e.g., treatment providers, recovery clinics, support groups). The principles of this app are grounded in Substance Abuse and Mental Health Services Administration (SAMHSA) clinical guidelines for SUD recovery, ensuring alignment with best clinical practices.

We used purposive sampling to recruit directly from facilities (i.e., treatment recovery centers, recovery homes, justice settings, and emergency rooms) across Missouri that engage individuals who use substances. We also employed snowball sampling techniques, a method that aids in reaching populations that are typically difficult to access (31), such as individuals with SUD (20, 32, 33). In this snowball sampling approach, previous clients referred new individuals to the uMAT-R study either via word of mouth, or through the distribution of our IRB approved physical or digital uMAT-R flyers among peers. Additionally, staff members at partner facilities also spoke with their clients about the study and distributed flyers to those who expressed interest in the study. Eligibility criteria for the study included having ever used opioids and/or stimulants, receiving treatment at one of the consenting facilities, being 18 years or older, being a U.S. resident, being fluent in English, and owning a smartphone with either an iOS or Android operating system. All participants had received a formal SUD diagnosis from their treatment facility and were actively engaged in substance use treatment at the time of their participation in the study. The study protocol was reviewed and approved by the university’s Institutional Review Board (IRB ID# 201910161). All participants provided informed consent for this study. After an initial review of the data, one case was omitted from the data analysis due to insufficient information provided during the interview.

### 2.2 Semi-structured interviews

For this study, individuals were invited to participate in a follow-up qualitative interview after completing the one-month uMAT-R mHealth intervention. Participants were interviewed individually by trained research staff; participants knew staff from the e-coaching role (described above) and in-person study recruitment events. All interviewers held a bachelor’s degree or higher in psychology or a related field and have received training in crisis management, active listening, and rapport-building. The research staff member that originally consented the participant to the study was not necessarily the assigned e-coach nor the interviewer. Each participant was compensated with a $10 gift card. Interviews ranged from 30 to 60 min long. Interviews were conducted using the phone feature of the Zoom video communication app and were audio-recorded. Interview transcripts were not returned to participants. If the participant declined to have their interview recorded (which was asked during the informed consent process, the interviewers took detailed notes). Interviewers asked questions from an approved interview guide. The complete interview guide included questions about participants’ personal experience seeking recovery, the benefits and challenges to participation in the intervention, and feedback about the uMAT-R mHealth app (see examples of select questions in Table 1). Interviewers occasionally asked probing or follow-up questions to explore and clarify participants’ responses. Demographic information was collected using a baseline web-based questionnaire. The interviewers uploaded the interview audio to BOX, a secure online platform and completed interview summaries after each interview to provide an audit trail of the qualitative research process and to ensure fidelity to the interview protocol and guide (34).

**Table 1:** Relevant semi-structured interview guide questions.

Parent questions
*What are worries/concerns/stressors that impact your recovery?*
*What helps you the most to stay sober/to commit to recovery? (e.g., certain practices, help-seeking behaviors).*
*What is the hardest part about recovery/staying sober?*
*How do you manage to take care of daily needs for you and your family? (e.g., buy groceries, pay for utilities, manage chores, work if relevant)*
*Do you have a supportive group of family members or friends that support your recovery?*
*Do you feel as though your use of substances has impacted your relationships with any of the individuals identified above? If yes, how so?*
*How does stigma impact you? Is stigma something that you experience? Stigma often involves experiencing negative attitudes or discrimination based on a specific characteristic.*

### 2.3 Qualitative data analysis

Audio recordings were transcribed verbatim by a university-approved service and reviewed for accuracy. The interview transcripts were analyzed using deductive thematic analyses, with the aim of extracting and thematizing participants’ recovery experiences. The authors took a positivist qualitative methodological stance so that findings provided a summary of topics from the dataset (35). Thematic analysis refers to a systematic and flexible qualitative method that can be applied across a range of epistemological and theoretical approaches (36).

Two research staff members coded the transcripts independently using a codebook initially based on the interview guide. The coders manually coded for the presence and absence of codes for each participant in an Excel workbook. The two primary coders were both cisgender female students working towards advanced degrees in social work and medicine. The principal investigator (PI) served as the third coder and is a cisgender female and doctoral-level licensed clinical social worker with expertise in behavioral and mental health. The PI identified coding discrepancies between the two coders and informed additional codebook development. For example, after the first pass of coding the transcripts, the team decided to add a code about substance use and the impact on relationships to the codebook. The team members addressed discrepancies in agreement among other codes and recoded the data using the updated codebook. Prior to team discussion, intercorder agreement was 90%. This measure involves calculating the number of agreements on given codes between the coders divided by the total number codes (37). The value is then multiplied by 100, which higher numbers suggesting higher agreement (38). After discussing and addressing coding discrepancies, the coding agreement improved to 94%. Finally, the team iteratively defined and refined the definitions of the final major and minor themes. Minor themes include themes that were less frequently mentioned among participants by topic. To determine code saturation, the team used both coding frequency and code meaning approaches (39, 40). The study’s sample of 24 interview transcripts allowed for sufficient analyses of themes, as 9–17 interviews are considered the benchmark for reaching code saturation among a fairly homogenous sample population (39, 40). Final themes (both major and minor) are in Table 2.

**Table 2:** Parent and subthemes with exemplar quotes.

Parent theme	Subthemes	
Worries and concerns (*n* = 12)	Money/finances/ being employed/working	*“…I wanna get back to work and start repaying my daughter back. That’s [a] really heavy burden.”*
	Cravings/returning to use^a^	*“Yeah, it’s my body basically telling me like, ‘Hey, go get the alcohol and put it in.’ It was almost like my body was saying that, but it was hard to explain…”*
	Self-medication for mental and physical pain^a^	*“Drug use or alcohol makes me chipper quicker sort of thing. It snaps me right out of that depression. I self-medicated quite a bit.”*
	Telling others about substance use^a^	*“I wasn’t gonna tell her ‘cause I thought first of all. Then, when I realized we are probably gonna hang out, I better tell her…”*
What helps with recovery (*n* = 24)	Accountability through providers/services	*“At this point, being in an environment that does[drug] test and that I come back to other people who are kind of in the same struggle…”*
	Loved ones/children	*“My kids, getting ‘em back.” (Custody lost due to substance use).*
	Being in treatment/medication	*“What’s helped me the most to keep me from drinking is medications and doctor’s visits where I’m actually taking medications.”*
	Not going back to the old life	*“The willingness to want to change and not to go back to my old ways.”*
	Daily practices^a^	*“Just relyin’ on my higher power, I guess. My motivation to stay sober and clean is I enjoy life a lot better.”*
Hardest part of recovery (*n* = 22)	Changing behavior/lifestyle	*“It’s a different lifestyle for me. Dealing with, I guess you’d say boredom, it’s gonna be hard.”*
	Missing the substance	*“I just miss it. It was just something fun for me to do until it became not fun. “*
	Navigating treatment system^a^	*“…finding the right treatment and then just the detox process… Ooh, Lordy. My gosh.”*
	Managing life stressors (emotional/physical)^a^	*“Well, a lot of the reason I used was because of physical and emotional pains.”*
	Managing relationships/ fallout of SUD^a^	*“My brother’s attitude towards me…It’s very, very hard.” (brother not believing that participant is in recovery)*
Taking care of daily needs (*n* = 24)	Treatment facility helps with basic needs	*“Right now, I do not have to worry about that because I’m in a sober living house. All I have to worry about is me.”*
	Government/subsidized services like food stamps help meet basic needs	*“I get TANF cash assistance on my food stamp card, so that’s how I buy stuff that I need for the baby. I get WIC, too…”*
	Being self-sufficient and trying to make ends meet for now	*“I set aside money and stuff that I know I’ll need a month from now. I’m really good at doing that.”*
	Family/ significant other helps with meeting basic needs^a^	*“Well, what helps is I do communicate with my girlfriend. I try to first—I try not to—I try not to overwhelm myself. I try to make a list of what I need and how to go about gettin’ whatever those needs are met.”*
Sources of support (*n* = 24)	Family/partners/friends	*“Yeah. I have a significant other…She’s supportive, but not to the point where—she will not do anything to enable me to put me in any kind of position.”*
	Peers	*“I have AA friends from where I live that I stay in contact with daily just in texting or if I need to bounce something off somebody.”*
	Treatment team/providers^a^	*“I was very diligent in the very beginning to work the steps, and make my amends, and do all the right things, and then I built up a network of clinicians, and support groups, and I kept seeking out more support because I did not have the family.”*
Substance use impacted relationships (*n* = 19)	Devastated/ruined relationships	*“I had lost all relationships with everybody. If I would continue, those people would not be in my life now.”*
	Trying to mend relationships now	*“…with my family, I’m gradually moving my way back into the group. They pretty much wrote me off.”*
	Pushing others away^a^	*“Oh, shit yeah. Towards the end, it was just drinkin’ and workin’ and drinkin’ and workin’, and that was it—or usin’ or whatever.”*
Stigma related to substance use (*n* = 20)	Being seen differently by others	*“My family definitely knows and yes, I get stigma from them.”*
	Being treated poorly by providers/social services	*“I’ve heard them in the other room saying, “Oh, she was a junkie. Why should we even bother trying to find a vein on her?”*
	Internalized stigma	*“…but I feel like people would look at me like I’m dirty, or like I’m a thief, and I’ll steal from them, or I’m not honest.”*

a Denotes minor sub-theme.

An established set of best practices for evaluating quality and rigor of qualitative research are inconclusive. For this study, we decided to evaluate quality by following the four criteria for trustworthiness based on the seminal work of Lincoln & Guba: credibility, transferability, dependability, and confirmability. Please see Appendix for details on how we evaluated each criterion (41, 42). Additionally, to enhance the study’s rigor, the analytical team identified and examined two negative cases. Negative cases are defined as those which deviate from the main theoretical perspective (43). Negative cases help to set limitations on a theme or to identify new aspects of a theme (44). One participant responded no to questions related to any difficulties or negative experiences related to recovery. Another participant shared that they had experienced stigma because of mental health needs only. The potential implications for these observations will be addressed in the discussion section.

## 3 Results

Twenty-four adults at risk of opioid-related overdose (e.g., had ever used opioids, stimulants, and/or hallucinogens) completed semi-structured interviews (see Table 3). Half of the sample (*n* = 12, 50%) was between the ages of 31–50 years old and most (*n* = 18, 75%) identified as female. The majority (*n* = 21, 87.5%) identified as white and Non-Hispanic/Latino (*n* = 24, 100%). About 71% (*n* = 17) stated that they were currently unemployed, and 87.5% (*n* = 21) disclosed that they were recipients of Medicaid. Twelve participants (50%) described stimulant use as their primary substance use issue, followed by opioid use (*n* = 10, 42%), and hallucinogen use (*n* = 2, 8%). Participants who described opioid use as their primary substance use issue were asked if they were taking MOUD and 70% (*n* = 7) responded yes. Among those participants, 6 were currently taking Buprenorphine and one person was taking Methadone.

**Table 3:** Participant demographic characteristics.

Characteristic	*n* (%)
Age (range)	
18–30	4 (17)
31–50	12 (50)
51+	8 (33)
Gender	
Male	6 (25)
Female	18 (75)
Race/Ethnicity	
White	21 (87.5)
African American	3 (12.5)
Hispanic (Yes/No)	
Yes	0 (0)
No	24 (100)
Highest level of education^a^	
10th grade completed	1 (4.1)
11th grade completed	1 (4.1)
Regular high school diploma	6 (25)
GED certificate of high school completion	4 (16.7)
Some college credit, but no degree	6 (25)
Associate’s degree (Ex.: AA, AS)	2 (8.3)
Bachelor’s degree (Ex.: BA, BS)	4 (16.7)
Employment status	
No, I am not employed	17 (70.8)
Yes, full-time	7 (29.2)
Current living arrangement^a^	
Homeless – shelter	1 (4.1)
Homeless – streets	1 (4.1)
Treatment facility/medical center	7 (29.2)
Living in someone else’s home/apartment	7 (29.2)
Living in my own home/apartment	8 (33.3)
Health insurance status^a^	
None	1 (4.1)
Medicaid	21 (87.5)
Private	1 (4.1)
Medicaid and private	1 (4.1)
Round of recovery	
1st time in recovery	7 (29.2)
2nd time in recovery	4 (16.7)
3rd time in recovery	6 (25)
4th time in recovery	3 (12.5)
5th time in recovery	1 (4.1)
More than 5 times	3 (12.5)
Primary substance use issue^a^	
Opioid use	10 (41.6)
Stimulant use	12 (50)
Hallucinogen use	2 (8.3)
MOUD (Yes/No)	
Yes	7 (29.2)
No	3 (12.5)
N/A	14 (58.3)

a Due to rounding, the sum of percentages does not exactly equal 100.

The following demonstrates the major and minor subthemes identified regarding the following topics: how to take care of daily needs, factors that help with recovery, sources of support, most difficult part of recovery, worries and concerns about recovery, the impact of substance use on relationships, and stigma because of substance use. Table 2 illustrates these themes and exemplar quotes from participants.

### 3.1 Worries and concerns

Half of the study participants (*n* = 12) expressed worries and concerns related to recovery. The most prominent theme included constant worries about money and finances and the challenge of finding work following treatment. Many participants mentioned that they wanted to pay loved ones back for financial support, and that they wanted to feel more self-sufficient. Three minor themes were identified. First, some participants had worries about managing cravings for substances, especially in scenarios where substances may be present, such as alcohol being served at a restaurant. Second, participants specifically noted how they had previously used substances to self-medicate for physical or emotional pain, and that they thought about how the substances had been beneficial during those times. Lastly, participants discussed the anxiety and anticipation of having to tell longtime friends and colleagues about their substance use and recovery status for the first time.

### 3.2 Factors that help with recovery

Every participant (*N* = 24) shared specific factors that help with recovery. Major themes included support from treatment providers and recovery services to focus on recovery, motivation for recovery to improve relationships with loved ones/children and remaining in treatment and/or staying medication adherent. A final major theme included not wanting to go back to the old way of life when using substances. Participants shared a minor theme of using daily practices to stay engaged in the recovery process and to rebuild a healthy and quality life, such as reading, studying for a degree, going on daily walks, or practicing religion and prayer.

### 3.3 Most difficult part of recovery

Twenty-two participants identified the hardest part of recovery for them. Major themes included learning how to change one’s behavior or lifestyle without substance use and yearning for the substance (e.g., memories of positive feelings tied to substance use).

Three minor themes were observed. First, participants reported barriers to recovery including difficulty of navigating the treatment and healthcare systems, specifically finding the correct facility for their needs. Second, participants mentioned the challenge of managing recovery during life stressors. Lastly, participants shared the difficulty of managing relationships (e.g., not turning to substances after a fight) and rebuilding trust.

### 3.4 Taking care of daily needs

Twenty participants discussed how they fulfill their daily needs (e.g., housing, clothing, food). Major themes included having their treatment facility take care of basic needs for the time being, the use of government and subsidized services and programs (e.g., The Women, Infants, and Children program (WIC)) to make ends meet and practicing self-sufficiency by budgeting or finding employment. A minor subtheme included having family or a significant other help with meeting basic needs. This included having the loved one financially support the participant or help them to stay accountable for daily tasks that needed to be met.

### 3.5 Sources of emotional and social support

All participants (*N* = 24) shared who they consider to be sources of emotional and social support for recovery. Major themes included loved ones/family and peers met during the recovery process. A minor theme included reliance on the treatment team and other providers for emotional support. A few participants explained how they no longer have the emotional and social support of family and friends and that providers have helped to fill some of this gap.

### 3.6 Impact on relationships

Nineteen participants disclosed the impact of their substance use on their relationships. Major themes included how substance use and actions when using ruined relationships and how they are working to mend many relationships now that they are in recovery. A minor theme included the loss of loved ones due to substance use consuming their life.

### 3.7 Stigma related to substance use

Twenty participants reported feelings of stigma due to their substance use. Major sub-themes included perceptions of being viewed differently by family and friends because of their past substance use and stigmatized interactions with providers based on their substance use history and current struggles. Participants also indicated internalized stigma that was anticipated when engaging with others.

## 4 Discussion

Within the context of the ongoing role of fentanyl in overdoses and poisonings (45, 46), and the lower receipt of SUD treatment among people in need, this study identified various interpersonal and intrapersonal factors that may play a critical role in recovery. Financial stressors, worries about daily needs, damaged social relationships, and stigma (either being the receipt of or internalized) were critical challenges that trigger cravings or recurrence to substance use, while healthy relationships with loved ones and peers and motivation for financial stability are critical factors that may shape success in recovery. This research contributes needed insight on the client experience, which can inform how providers problem solve barriers to treatment while emphasizing factors that promote treatment engagement. The study also highlights the necessity of recovery-oriented approaches to care, as findings indicate which factors may be better handled by a peer or counselor within a recovery service or treatment setting.

Most participants described challenges related to substance use and recovery, and many relied on the treatment team or loved ones for accountability and modifying destructive behaviors. The majority of participants reported concerns regarding access to employment and financial independence after treatment, which aligns with recent findings that participants with a history of a substance use disorder were less likely to be employed or retired than the general population (47). We identified one participant who did not have worries or concerns about recovery. While this person may feel prepared for handling recovery, their response may also reflect a hesitancy to consider what may go wrong. Taken together, our findings demonstrate to the field that providers should seize the opportunity to discuss and plan for independence and boost personal agency with clients, while being mindful of a person’s fear of considering future setbacks.

Amidst challenges, participants described motivation to make the most of treatment, and how they can work towards successfully caring for themselves by budgeting and using public programs. Many participants stated an eagerness to repay loved ones for financial support or for funds misused when they had been using. Treatment teams may refer clients to recovery RCCs that can offer support with job readiness. Interventions such as the Customized Employment Supports (CES) model (48) and the Vocational Problem-Solving Skills (VPSS) intervention (49) have shown promise in helping individuals with substance use issues obtain employment and improve employment functioning. Previous research has demonstrated that access to employment is one of the major predictors of positive treatment outcomes and sustained engagement in recovery (50, 51).

Many participants identified their relationship with loved ones, especially their children, as a significant factor that helps with recovery and motivates progress. They likewise mentioned peers from treatment or at mutual support groups as a major source of social support. Such groups offer hope and motivation in their recovery journey. This is emphasized by available research, which demonstrates emotional support from loved ones and from peers may be vital to staying engaged in substance use treatment (40, 52, 53). Individuals who attended RCCs have given high ratings of recovery support groups in terms of perceived helpfulness, suggesting those early in recovery may benefit from interactions with peers and these interactions may help to foster a sense of belonging that can decrease feelings of shame and self-stigma (54). Thus, RCCs are may be ideal settings to reinforce the importance and usefulness of peer support and strengthening relationships with loved ones in achieving and maintaining recovery.

At the same time, our participants experienced significant stigma surrounding substance use from family, friends, and care providers, which is consistent with prior research (55–57). One participant reported that they had only experienced stigma specific to mental health issues, which suggests that individuals are vulnerable to different types of discrimination when seeking help. Our findings add to the literature surrounding awareness that stigma is a major barrier in the recovery process (58). Healthcare systems have the potential to mitigate providers’ attitudes towards substance-use and adults in recovery, but such strategies can be complex and require a nuanced approach (59). Prior research has indicated that successful components of stigma reduction include the use of “person-first” language, emphasis of substance use as a treatable condition, utilization of messaging with sympathetic narratives, and emphasis of societal rather than individual causes of addiction (60). Providers may develop more opportunities for loved ones to receive psychoeducation about substance use and recovery, where they can also receive support as a caretaker, parent, or partner.

Our findings also suggest that individuals experience emotional turmoil about past social relationships that can impede recovery. Studies show that loss of identity and relationships with others are common during addiction recovery (61) and may result in shame and guilt (62). Our study is impactful for signaling that individuals in recovery may benefit from discussions on how to process past experiences, how to rebuild or develop new relationships, and how to develop a quality life without substances. Our results stress the need for guidance on how to view oneself beyond substance use.

Demographic characteristics lend to the current psychosocial strengths of the sample’s individuals while highlighting factors that may test the recovery process. For example, most participants had at least a high school diploma but were currently unemployed and insured through Medicaid. About 70% of our sample was interviewed in at least their second instance of substance use treatment. At the time of sampling, about 33% of participants lived in their own home, while about 58% were living in someone else’s home or at a treatment facility. Most participants with a primary diagnosis of OUD were currently taking MOUD to support recovery.

Lastly, it is important to acknowledge that loved ones and family appeared in multiple major and minor subthemes. As demonstrated by these themes, loved ones and family could provide resources such as housing, and financial support, or emotional and social support. At the same time, they could be a source of anxiety and stress for a client when anticipating how they would react to finding out about the person’s SUD for the first time. Interactions among loved ones and family could also feel stigmatizing once the client had become identified socially as an individual with an SUD. Thus, the multiple roles that loved ones and family members could play in a client’s recovery demonstrate how complicated it must feel internally to navigate relationships and the feelings that they evoke. Providers may find value in asking about the purpose and nature of client’s relationships beyond only assessing for the presence or absence of supports in general.

### 4.1 Limitations

Our sample was restricted to participants living in Missouri. However, considering the needs of our patient population (e.g., individuals seeking substance use treatment in a largely rural and under-resourced state), findings may have transferability to similar states and communities. Our sample was primarily female and white, with may be attributable to recruitment methods. The female-predominance in our sample may be attributable to the snowball sampling strategy, as many treatment settings serve only one gender or may otherwise limit interactions among people of different genders. The high proportion of white participants reflects both a state-specific and national disparity in substance use treatment engagement for Black Americans, largely attributable to enduring institutional and societal barriers (e.g., racism, non-culturally appropriate services) (8, 62).

There is the possibility that social desirability bias may have influenced participants’ responses, especially as some e-coaches also served as interviewers. To mitigate this, participants were reminded that they can stop the interview or decide to not answer certain questions. The research team also noted that established trust between the participant and e-coach helped to create a more comfortable interview experience for the individual and promoted more active discussion. To prevent investigator bias on the study findings, the data collection team differed from the analytical team.

## 5 Conclusion

Overall, these findings emphasize a nuanced and evolving recovery experience among individuals who are at risk of opioid-related overdose, which may be less evident in interactions with clinical treatment. Our study highlights the strengths of people in recovery, such as taking care of basic needs, changing destructive behaviors, and utilizing treatment and medication. Clients will likely benefit from additional guidance in preparing for life after substance use treatment, such as rebuilding relationships and finding new social supports, and navigating pervasive stigma. Services that offer a recovery-oriented approach may be opportune settings for clients to have non-clinical needs met.

## Data Availability

The datasets presented in this article are not readily available because the dataset will not be shared as raw, anonymized data may include information that could be identifiable and the information is sensitive. Requests to access the datasets should be directed to szlyk@wustl.edu.

## Appendix

**Table A1:** Evaluation of study quality and rigor.

Criteria	Strategy	Study examples
Credibility	Prolonged engagement	Participants had met interviewers previously at an in-person recruitment event; some participants may have had their e-coach as their interviewer.
	Reflexivity	Interviewers often checked in with principal investigators about interviewing experience and emerging perceptions. The third coder did not conduct any of the interviews and could obtain a more objective stance during analysis; co-authorship team members were not all research team members.
	Triangulation	After every interview, the interviewer completed an interview summary template. Each participant also completed surveys about themselves and their recovery process.
Transferability	Thick descriptions	Authors provided detailed descriptions of the parent study, the study setting, and the data collection and analysis process. Readers can determine if themes are transferable to their client population.
	Sampling strategies	The authors used purposive and snowball sampling, which also mirrors how potential clients are engaged in behavioral services in the region.
Dependability	Methodological documentation	The procedure of the study is documented in the methods section of the manuscript.
	Audit trails	Each interviewer completed an interview summary after each interview, which recorded overarching themes of the interview, how the interview compared to others, and points to consider for future interviews. The principal investigator kept separate notes to document the coding process.
Confirmability	Peer debriefing	The two primary coders and the third coder discussed any disagreements during the initial and final coding process. The process of intercoder agreement also demonstrated which specific topics required more discussion among the coding team. Results were shared with colleagues and feedback was integrated into the final themes.
